# Evaluation of Safety and Antigenotoxic Activity of *Rosa centifolia* Extract, Kaempferol, and Kaempferol-3-glucoside against Ultraviolet B Radiation in Human Fibroblasts

**DOI:** 10.1021/acsomega.5c08600

**Published:** 2025-12-01

**Authors:** Silvia Ximena Barrios Martínez, Lady Johanna Sierra Prada, Raquel Elvira Ocazionez, Elena E. Stashenko, María Pilar Vinardell, Jorge Luis Fuentes

**Affiliations:** † Laboratorio de Microbiología y Mutagénesis Ambiental (LMMA), Grupo de Investigación en Microbiología y Genética, Escuela de Biología, Facultad de Ciencias, 28014 Universidad Industrial de Santander (UIS), Bucaramanga 680001, Colombia; ‡ Centro de Investigación en Biomoléculas, CIBIMOL, Facultad de Ciencias, Universidad Industrial de Santander (UIS), Bucaramanga 680001, Colombia; § Laboratorio de Cromatografía, CROM-MASS, Escuela de Química, Facultad de Ciencias, Universidad Industrial de Santander (UIS), Bucaramanga 680001, Santander, Colombia; ∥ Departamento de Bioquímica y Fisiología, Facultad de Farmacia y Ciencias de la Alimentación, 16724 Universitat de Barcelona, Barcelona 08028, Spain

## Abstract

The plants can be a source of compounds that prevent UV-induced DNA damage involved in the genesis of skin cancer and aging. This work was aimed to evaluated the safety and the antigenotoxic effect of *Rosa centifolia* flower ethanolic extract and of selected flavonoid constituents against UVB radiation in MRC-5 human fibroblasts. The cytotoxicity and genotoxicity of the phytochemicals were evaluated using trypan blue exclusion and Comet assays, respectively. The assays revealed that *R. centifolia* extract, kaempferol, kaempferol-3-glucoside, and quercetin exhibited cytotoxic effects at concentrations of 363 μg/mL, 393 μM, 379 μM, and 141.1 μM, respectively. Additionally, *R. centifolia* extract and quercetin demonstrated genotoxic effects at the highest tested concentrations. The antigenotoxic effects of *R. centifolia* extract, kaempferol, and kaempferol-3-glucoside against UVB radiation were subsequently evaluated. These phytochemicals significantly reduced UVB-induced DNA damage in human fibroblasts at noncytotoxic concentrations. Therefore, these compounds represent promising candidates for sunscreen formulations for human photoprotection.

## Graphical abstract

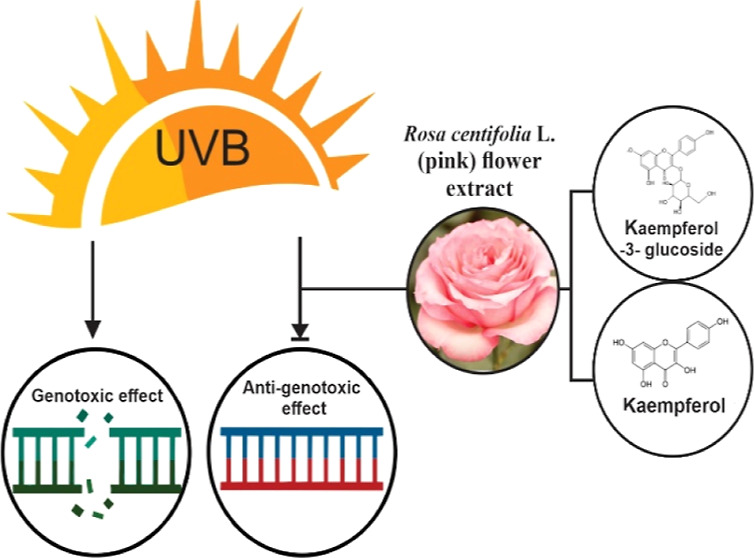

## Introduction

Solar radiation absorbed by human skin cells can induce DNA chemical modifications, resulting in mutations and subsequently malignant cellular transformations.^1,2^ Plant-derived compounds represent a source of pharmacologically safe agents for human skin photoprotection.^3^ These compounds prevent solar radiation-induced mutagenesis, which is implicated in skin cancer.^4,5^ Therefore, the use of sunscreen improved with plant-derived compounds is now a common photoprotective practice to mitigate solar radiation skin damage.^6–9^

*Rosa centifolia* is a widely used plant species due to its cosmetic and medicinal properties.^10–12^ Several studies have demonstrated diverse *in vitro* and *in vivo* bioactivities of *R. centifolia* extracts, with the most relevant properties including antioxidant and antidiabetic,^13^ anti-inflammatory and antiarthritic,^14^ antiaging,^15^ and protective effects against UV radiation-induced damage.^16^ Chemical characterization of *R. centifolia* shows the presence of carbohydrates, flavonoids, phenolic acids, tannins, and terpenoid compounds.^11,12^ Using UHPLC–ESI^+^–Orbitrap–MS, we have identified several major flavonoid compounds (e.g., kaempferol, kaempferol-3-glucoside, kaempferol-rhamnoside, quercetin, and quercetin-3-rhamnoside) in a *R. centifolia* flower ethanolic extract.^16^

Flavonoids are multifunctional molecules known for their photoprotective potential, which can act as antioxidants, UV-filters, anti-inflammatories, and immunomodulatory agents.^17,18^ Some reports^19–21^ have confirmed that the flavonoids kaempferol, kaempferol-3-glucoside, and quercetin have anti-inflammatory effects, which is an activity relevant for protecting skin against UV-induced damage. In addition, flavonoids kaempferol and quercetin have demonstrated photoprotective activity,^22^ while kaempferol also showed antigenotoxicity activity against UVB radiation.^23^ However, this bioprospection antigenotoxicity study was developed using a bacteria-based assay, and the mode of action of the antigenotoxic substances should be studied in target human cells or mammalian models where the drug will be used.^24^

The present study aimed to evaluate the cytotoxicity and genotoxicity of the *R. centifolia* flower ethanolic extract and of its major constituents (kaempferol, kaempferol-3-glucoside, and quercetin) in human fibroblast cells. Additionally, the antigenotoxic properties of these phytochemicals against UVB-induced DNA damage were evaluated.

## Results and Discussion

The present study focuses on the potential utility of an ethanolic extract obtained from *R. centifolia* flowers for human skin photoprotection. The flavonoid-rich chemical composition identified in this *R. centifolia* extract (Figure S1) was consistent, at least partially, with previous reports on *R. centifolia*,^12^ but differed from those previously reported for their flower essential oils.^11^ These differences reflect the distinct extraction methodologies and the chemical nature of the targeted metabolites. Distillation preferentially isolates volatile monoterpenes, whereas solvent extraction recovers polar compounds, such as flavonoids and phenolic acids.

The cytotoxic effects of *R. centifolia* extract and the three commercially available constituents (kaempferol, kaempferol-3-glucoside, and quercetin), which represented ∼75% of the mass (mg/g) of this extract (Figure S1), were assessed in human fibroblast cells (Figure 1). The *R. centifolia* extract and tested compounds induced significant reduction in fibroblast viability coming from the following concentrations: *R. centifolia* extract (500 μg/mL), kaempferol-3-glucoside (223 μM), kaempferol (87 μM), and quercetin (21 μM). Using graphical interpolation methodology,^25^ the concentration producing 50% (CC_50_) and 30% (CC_30_) of cell cytotoxicity in human fibroblasts were determined. The cytotoxicity CC_50_ values were as follows: *R. centifolia* (492 μg/mL), kaempferol (1398 μM), kaempferol-3-glucoside (676 μM), and quercetin (222 μM), comparable to those previously reported by us.^16,22,26^ These phytochemicals were innocuous to fibroblast cells at concentrations ≤ CC_30_ values as follows: *R. centifolia* (363 μg/mL), kaempferol (393 μM), kaempferol-3-glucoside (379 μM), and quercetin (141.1 μM).

**Figure 1:**
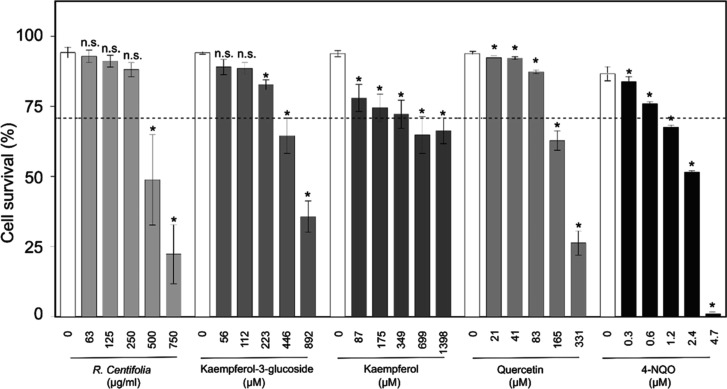
Cytotoxicity of the studied phytochemicals in MRC5 human fibroblasts. The mutagen 4-nitroquinoline 1-oxide (4-NQO) was used as a positive control (PC). Survival data were expressed as the average values and their corresponding standard errors (SEM), calculated from three independent experiments (*n* = 3). *, Significant (*p* < 0.05) differences with respect to negative control. n.s. Not significant differences.

Genotoxicity dose–response relationships were investigated in human fibroblasts for each phytochemical (Table 1). In accordance with previous studies,^16,26^ *R. centifolia* extract and quercetin exhibited dose-dependent genotoxicity with relevant genotoxicity (genetic damage index (GDI) ≥2) at the highest tested concentrations of 750 μg/mL and 225 μM, respectively. In a previous study,^27^ kaempferol induced minimal DNA damage at 874 μM, whereas kaempferol-3-glucoside exhibited non-genotoxic effects at any tested concentration. All genotoxic phytochemicals showed significant positive correlations between the concentration and genotoxicity, confirming dose–response relationships. Based on previous findings,^28–30^ and the present results regarding quercetin’s cytotoxicity and genotoxicity, quercetin was excluded from subsequent antigenotoxicity studies.

**Table 1:** Genotoxicity of the Studied Phytochemicals in MRC5 Human Fibroblasts^a^ ^,^ ^b^

*R. centifolia* extract		Kaempferol-3-glucoside		Kaempferol		Quercetin	
Conc. (μg/mL)	GDI ± SEM^c^	Conc. (μM)	GDI ±SEM	Conc. (μM)	GDI ±SEM	Conc. (μM)	GDI ±SEM
0	0.20 ± 0.05	0	0.24 ± 0.07	0	0.21 ± 0.03	0	0.09 ± 0.01
47	0.34 ± 0.07	44	0.15 ± 0.11	109	0.38 ± 0.06	14	0.45 ± 0.13
94	0.38 ± 0.09	89	0.19 ± 0.03	218	0.48 ± 0.12	28	0.64 ± 0.06
188	0.43 ± 0.15	176	0.22 ± 0.08	349	0.55 ± 0.06	56	0.87 ± 0.18
250	1.11 ± 0.18	352	0.29 ± 0.08	437	0.71 ± 0.18	113	1.27 ± 0.15
375	1.10 ± 0.34	376	0.35 ± 0.16	874	1.27 ± 0.07	225	2.15 ± 0.21
750	2.62 ± 0.35	702	0.28 ± 0.09	1747	1.30 ± 0.10		
PC	3,91 ± 0,01	PC	3.99 ± 0.02	PC	3.62 ± 0.02	PC	3.85 ± 0.07
*R* = 0.97 (*p* < 0.05^d^)		*R* = 0.65 (*p* = 0.1, n.s.)		*R* = 0.87 (*p* < 0.01)^d^		*R* = 0.98 (*p* < 0.05^d^)	

a The GDI average values and their corresponding standard errors (SEM) calculated from at least three independent experiments (*n* = 3) are given. The Pearson correlation coefficient (R) between GDI values and extract concentrations was also shown.

b The standard mutagen 4-nitro-quinoline-1-oxide (0.89 μg/mL) was used as a PC.

c The DNA damage criteria were as follows: (i) GDI values between 0 and 1 (no DNA damage), (ii) GDI values between 1 and 2 (little DNA damage), (iii) GDI values between 2 and 3 (moderate DNA damage), and (iv) GDI values between 3 and 4 (severe DNA damage).

d Significant correlation, n.s., no significant correlation.

The present results demonstrated that *R. centifolia* flower extract exhibits dose-dependent cytotoxicity and genotoxicity in human fibroblasts, consistent with previous results in other *Rosa* species.^31,32^ At least two major compounds (kaempferol and quercetin) present in the ethanolic extract, which comprised 61% of the extract mass (mg/g), resulted in mild to moderate cytotoxic and genotoxic effects in human fibroblasts, respectively. Kaempferol has been shown to induce apoptosis in human cervical cancer HeLa and SiHa cells,^33,34^ while quercetin resulted in cytotoxicity to human hepatoma HepG2 cells,^28^ increased chromosomal aberration in genetically engineered V9 Chinese hamster lung fibroblast,^29^ and elevated sister chromatid exchange and micronuclei in Chinese hamster ovary cells.^30^ These findings support the hypothesis that quercetin largely contributed to the extract’s cytotoxicity and genotoxicity at higher concentrations. Our results evidence that *R. centifolia* ethanolic extract is safe at concentrations ranging from 47 to 345 μg/mL; however, these results also emphasize the necessity of establishing safe concentration thresholds for incorporating these phytochemicals into cosmetic or sunscreen formulations.

In a previous study,^27^ we demonstrated that UVB radiation dose at 87.5 mJ/cm^2^ induced significant DNA damage in human MRC5 fibroblast cells while maintaining cell viability above 70%. Accordingly, this radiation dose was selected for antigenotoxicity studies of the phytochemicals. Here, we demonstrated that the *R. centifolia* flower ethanolic extract, kaempferol, and kaempferol-3-glucoside inhibited UVB-induced DNA damage in human fibroblasts. Results for antigenotoxicity against UVB radiation of the phytochemicals are shown in Table 2.

**Table 2:** Antigenotoxic Effects against UVB Radiation of *Rosa centifolia* Extract and Their Major Constituents in MRC5 Human Fibroblast Cells^a^

*R. centifolia* (pink)		Kaempferol-3-Glucoside		Kaempferol	
Conc. (μg/mL)	GDI ±SEM (%GI)	Conc. (μM)	GDI ±SEM (%GI)	Conc. (μM)	GDI ±SEM (%GI)
0	0.20 ± 0.05	0	0.24 ± 0.07	0	0.21 ± 0.03
47	1.00 ± 0.22 (65%)^b^	44	0.45 ± 0.15 (86%)^b^	109	0.88 ± 0.20 (73%)^b^
94	0.79 ± 0.30 (72%)^b^	89	0.62 ± 0.30 (81%)^b^	218	1.74 ± 0.60 (46%)^b^
188	0.60 ± 0.07 (79%)^b^	176	0.30 ± 0.06 (91%)^b^	349	1.02 ± 0.41 (53%)^b^
250	1.83 ± 0.08 (36%)^b^	352	0.28 ± 0.06 (91%)^b^	437	2.56 ± 1.35 (4%)^b^
375	1.87 ± 0.29 (34%)^b^	376	0.40 ± 0.06 (86%)^b^	874	2.65 ± 1.65 (12%)^c^
750	2.93 ± 0.14 (3%)^c^	702	0.37 ± 0.09 (89%)^b^	1747	3.00 ± 1.67 (16%)^c^
PC (87.5 mJ/cm^2^)	2.85 ± 0.51	PC (87.5 mJ/cm^2^)	3.27 ± 0.57	PC (87.5 mJ/cm^2^)	3.24 ± 0.81

a The GDI average values and their corresponding standard errors (SEM) calculated from at least three independent experiments (*n* = 3) are given. The percentage of genotoxicity inhibition (%GI) indicates the DNA damage protective effect with respect to PC treated with UVB radiation.

b Significant DNA damage reduction (%GI) with respect to PC.

c not significant differences.

*R. centifolia* extract significantly reduced UVB-induced DNA damage at concentrations ranging from 47 to 375 μg/mL, while kaempferol demonstrated protective effects at concentrations between 109 and 437 μM. This protective effect diminished at higher extract concentrations where these phytochemicals exhibited some degree of cytotoxic and genotoxic effects (see Figure 1 and Table 1). In contrast, kaempferol-3-glucoside demonstrated potent inhibition of UVB-induced DNA damage, reducing UVB-induced genotoxicity by 81–91% across all tested concentrations. These findings support previous studies on the antigenotoxic effects against UVR radiation of *R. centifolia* flowers extract observed in the SOS chromotest.^16^ *R. centifolia* hydrosols also resulted in antigenotoxic against the mutagen *N*-methyl-*N*-nitro-nitrosoguanidine (NMNG) in human lymphocytes.^32^ A recent review^12^ indicated that *R. centifolia* exhibits potent antioxidant, anti-inflammatory, and photoprotective properties attributed to its rich composition of compound flavonoids, phenolic acids, and tannins. For example, the *R. centifolia* aqueous extract showed anti-inflammatory and antiarthritic activity,^14^ while their hydroalcoholic extract exhibited antihyaluronidase and antioxidant properties.^15^

The major compounds of the *R. centifolia* ethanolic extract here studied (e.g., kaempferol and kaempferol-3-glucoside), especially the second one, showed a potent inhibitory effect of UVB-induced DNA damage. Previous studies^20,33–36^ showed that kaempferol has antioxidant, anticancer, and anti-inflammatory properties, and it also inhibits skin fibrosis in systemic sclerosis. Recently, we also showed that this compound has antierythema activity and stimulated DNA damage repair postirradiation in mouse skin.^27^ This study postulated that the observed antigenotoxic effects of this compound may be related to or possibly will be linked to inhibition of cyclobutene pyrimidine dimer (CPD) formation, the removal of CPDs, and restoration of normal cell division. On the other hand, kaempferol-3-glucoside showed antitumoral^37^ and anti-inflammatory activities^19,20^ and also attenuated UVB radiation-induced actinic keratosis formation.^38^ Together these findings are highly relevant for protecting against UVB-induced damage. These compounds not only preserve DNA integrity but also modulate inflammatory and apoptotic responses. Although speculative, we suggest that these flavonoids reduce UV-induced DNA damage by interfering with CPD formation, stimulating CPD repair, and reducing cellular inflammation. In fact, the inflammation (e.g., erythema action spectra) was closely correlated with CPD and UV genetic fingerprints induced by UVB (280–320 nm) and UVA^II^ (320–340 nm) spectral zones.^39^

Here, we showed a plant species promising as a source of sunscreen ingredient compounds; but as we indicated above, our results emphasize the necessity of establishing safe concentration thresholds for incorporating phytochemicals into cosmetic or sunscreen formulations. The findings demonstrating the safety profile of kaempferol-3-glucoside (e.g., no cytotoxic or genotoxic effect) in human epidermal cells support its high potential as an active ingredient in cosmetic and sunscreen formulations. This result was consistent with previous observations of plant compounds (e.g., apigenin, naringenin, and pinocembrin) that demonstrated protective effects against UVB-induced DNA damage.^40^ However, our data were based on fibroblast cell assays; therefore, at least a second evaluation in different skin cell models (e.g., keratinocytes and melanocytes) and/or a mammal model is recommended before these compounds are used for skin photoprotection. In addition, developing sunscreens based on these compounds will require the establishment of cost-effective processes to ensure a stable supply of these bioactive raw materials.

## Conclusions

The *R. centifolia* flower ethanolic extract and its constituent compound quercetin exhibit low to moderate cytotoxicity and genotoxicity at higher tested concentrations. These findings clearly evidence the necessity of establishing safe concentration thresholds for incorporating these phytochemicals in cosmetic or sunscreen formulations. At neither cytotoxic nor genotoxic concentration, the *R. centifolia* flower ethanolic extract and its constituent compounds kaempferol and kaempferol-3-glucoside demonstrated significant protective activity against UVB-induced DNA damage. These phytochemicals, particularly kaempferol-3-glucoside, which showed complete safety in human fibroblasts, appear promising as active ingredients in cosmetic and sunscreen formulations for human photoprotection.

## Materials and Methods

### Plant Extract

*R. centifolia* L. (pink variety) flowers obtained from Flexport–Colombia S.A.S. (Bogotá, Cundinamarca, Colombia) were used. Intact, fully mature flowers were lyophilized using an Advantage Plus Tray Lyophilizer (Virtis Co., Gardiner, ME, USA). The dried flowers were subsequently subjected to solvent extraction according to previously described methodology.^41^ In brief, dried flowers (1 g) were mixed with an acidified ethanol solution (20 mL, 0.5% HCl, 1:1 v/v) and put for 5 min in an S15H ultrasound bath (Elmasonic, Singen, Germany). The mixture was filtered, and the residue was extracted twice more. Extracts were rotoevaporated and then were dried as indicated above. Extract stock solutions were prepared from the dried powder (30 mg), which was dissolved in methanol (1 mL), vortexed (3 min), exposed to ultrasound (10 min, 40 °C), and centrifuged (5000*g*, 10 min). The supernatant (1 mL) was then filtered and was stored at −80 °C in a Thermo Scientific Series-86 DEG C ultralow-temperature freezer (Thermo Scientific, Waltham, MA, USA). The yield of the obtained hydroalcoholic flower extract was of 18.2 ± 0.1. Before their use, the extract stock solutions were defrosted and refrigerated (5–8 °C) for 24 h. Details on the chemical composition of this extract were previously obtained by us using UHPLC–ESI^+^–Orbitrap–MS,^16^ and this is summarized in Figure S1.

### Chemicals, Culture Media, and Solvents

The Bioultra lyophilized proteinase K, high-resolution agarose, trypan blue solution (0.4%), and the flavonoid compounds (kaempferol, kaempferol-3-glucoside, and quercetin) were obtained from Sigma-Aldrich Corporation (Milwaukee, WI, USA). The YOYO solution was purchased from Thermo Scientific (Waltham, MA, USA). The Dulbecco’s modified Eagle medium high glucose (DMEM-HM), fetal bovine serum (FBS), phosphate-buffered saline, trypsin EDTA solution, and antibiotics (penicillin–streptomycin mixture) were purchased from Gibco (Grand Island, NY, USA). The remaining reagents and solvents were purchased from J.T. Baker (Phillipsburg, NJ, USA) or Merck (Kenilworth, NJ, USA).

### Cytotoxicity and Genotoxicity Assessment of the Plant Extract and Compounds in Human Fibroblasts

Human fibroblast (MRC5) cells were graciously provided by Dr. Carlos Frederico Martins Menck from the Universidade de Sao Paulo (Sao Paulo, Brazil). The cytotoxic effects of the phytochemicals were evaluated in MRC5 human fibroblast cells using the trypan blue exclusion method as previously described.^23^ Briefly, cells were cultured in 5 mL of DMEM-HM supplemented with 10% FBS and 1% penicillin–streptomycin. Incubation was carried out at 37 °C in a humidified atmosphere with 5% CO_2_ using a Thermo Scientific Midi 40 incubator (Marietta, OH, USA). To maintain optimal growth, the medium was replaced every 3 days until cultures reached approximately 80% confluence. Cell cultures were exposed to various concentrations of *R. centifolia* extract, ranging from 63 to 750 μg/mL, kaempferol from 87 to 1398 μM, kaempferol-3-glucoside from 56 to 892 μM, and quercetin from 21 to 331 μM. The extract concentrations were mainly selected based on photoprotective relevancy,^16^ while the compound concentrations were selected both based on their solubility^42^ and photoprotective relevancy.^23^ The mixtures were then incubated under the previously described conditions (37 °C for 24 h under a 5% CO_2_ atmosphere). Cells cultured in DMEM-HM were considered the negative control, while the mutagen 4-nitroquinoline 1-oxide served as a PC. At least three independent experiments were carried out for each treatment. After 24 h, the cells were recovered, their viability was assessed using a Neubauer chamber, and their morphology was observed using an Eclipse E200 optical microscope (Nikon Instruments Inc., NY, USA). The CC_50_ and CC_30_ (50% and 30% cytotoxic concentrations) were calculated for each sample using a graphic method.^25^ Samples were considered cytotoxic at values ≥ CC_50_ and noncytotoxic at values ≤ CC_30_.

Genotoxicity analysis was conducted in MRC5 human fibroblast cells using the high-throughput Trevisan CometChip platform (Gaithersburg, MD, USA) and following a previously established protocol.^26^ Genotoxicity of the phytochemicals was assessed at concentrations ranging from 47 to 750 μg/mL for *R. centifolia* extract, 109 to 1747 μM for kaempferol, 44 to 702 μM for kaempferol-3-glucoside, 14 to 225 μM for quercetin, and 4.2 μM for the mutagen 4-nitroquinoline 1-oxide. DNA damage was quantified using the categorized approach, wherein comets were classified into five categories (0–4) according to Collins et al.^43^ The GDI for each treatment was subsequently calculated using the formula established by Pitarque et al.:^44^ GDI = (N_0_ × 0 + N_1_ × 1 + N_2_ × 2 + N_3_ × 3 + N_4_ × 4)/n, where N_*i*_ represents the count of nuclei in each respective category and *n* denotes the total number of cells evaluated per slide. GDI values were interpreted as follows: 0–1 indicated no DNA damage, 1–2 signified little DNA damage, 2–3 suggested moderate DNA damage, and 3–4 denoted severe DNA damage. Average GDI values for each treatment were derived from at least three independent experiments. For each treatment, 200 cells per slide were analyzed across two slides.

### Antigenotoxicity Assessment of Plant Extract and Compounds against UVB Radiation in Human Fibroblasts

Antigenotoxicity assays were also conducted in MRC5 human fibroblast cells employing the high-throughput Trevigen CometChip platform (Gaithersburg, MD, USA). The protective effect of phytochemicals against UVB radiation-induced DNA damage was assessed through cotreatment (cells exposed to UVB at 87.5 mJ/cm^2^ concurrently with phytochemicals). Cell irradiation was conducted as previously indicated.^27^ Briefly, the fibroblast mixture with phytochemicals (3 mL) was put into a Petri plate (5 cm diameter) and irradiated in darkness using an irradiation chamber BS-02 (Opsytec Dr. Gröbel GmbH, Ettlingen, Germany). This chamber was equipped with four-lamp UVB (280–315 nm) and four-lamp UVA (315–400 nm) in an intercalate configuration to achieve the highest irradiance for these two spectral regions. The chamber permits time- or dose-controlled irradiation of samples using the UV radiation controller UV-MAT. Two UVB and UVA sensors, calibrated with respect to the Physikalisch-Technische Bundesanstalt (Berlin, Germany), were used to measure UVB irradiance with the dose controller UV-MAT. The UV dose controller continuously measures irradiance, calculates doses, and stops irradiation at the set target dose. The concentrations of the tested samples were identical with those used in the genotoxicity studies. A UVB radiation dose of 87.5 mJ/cm^2^ was consistently used as a PC, while negative controls (nonirradiated cells) were included in all experiments. Subsequent procedures, including enzymatic lysis, electrophoresis, and genetic damage assessment, were performed essentially as previously described. Antigenotoxicity, defined as the capacity of a phytochemical to reduce UVB-induced genotoxicity, was determined by significant reductions in GDI values following coincubation.

### Statistical Analysis

The mean values of cell survival, GDI and %IG, and the corresponding standard errors were calculated. The Kolmogorov–Smirnov test and F-maximum test were applied to assess normality and variance homogeneity, respectively. Since the data passed these tests, the groups (treatment) were compared using a parametric Tukey’s test. The relationship between variables (compound concentrations and GDI estimates) was examined by using correlation analysis. A value of *p* < 0.05 indicated significance. The R platform^45^ was used for all analyses.

## Supplementary Material



## Notes

Major constituents identified in *R. centifolia* (pink variety) flower extracts using UHPLC–ESI+–Orbitrap–MS (PDF)
