# Supplementary material for: Evaluation of Safety and Antigenotoxic Activity of Rosa centifolia Extract, Kaempferol, and Kaempferol-3-glucoside against Ultraviolet B Radiation in Human Fibroblasts
Source: ACS Omega. 2025 Dec 1;10(48):59261–7. doi: 10.1021/acsomega.5c08600 (PMC12771157; doi:10.1021/acsomega.5c08600)
Supplement: Supplementary file 1 [file ao5c08600_si_001.pdf]

Evaluation of Safety and Antigenotoxic Activity of *Rosa centifolia* Extract, Kaempferol, and Kaempferol-3-glucoside Against Ultraviolet B Radiation in Human Fibroblasts

Silvia Ximena Barrios Martínez[a], Lady Johanna Sierra Prada[b], Raquel Elvira Ocazionez[c], Elena E. Stashenko[b,c], María Pilar Vinardell[d], and Jorge Luis Fuentes*[a,b]

[a] Laboratorio de Microbiología y Mutagénesis Ambiental (LMMA), Grupo de Investigación en Microbiología y Genética, Escuela de Biología, Facultad de Ciencias, Universidad Industrial de Santander (UIS), Bucaramanga, Colombia (jfuentes@uis.edu.co)

[b] Centro de Investigación en Biomoléculas, CIBIMOL, Facultad de Ciencias, UIS, Bucaramanga, Colombia

[c] Laboratorio de Cromatografía, CROM-MASS, Escuela de Química, Facultad de Ciencias, UIS, Bucaramanga, Santander, Colombia.

[d] Departamento de Bioquímica y Fisiología, Facultad de Farmacia y Ciencias de la Alimentación, Universitat de Barcelona, Barcelona 08028, Spain.

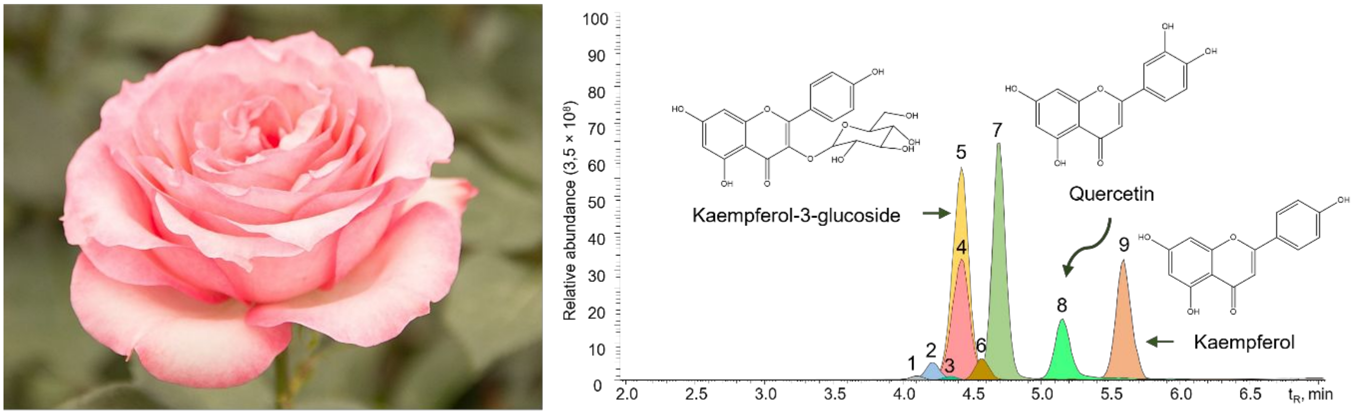

Figure S1. Major constituents identified in *R. centifolia* (pink variety) flower extracts using UHPLC–ESI+–Orbitrap–MS. Extract constituents (1 – 9) depended on their retention times (min) and their corresponding mass (mg/g) are shown as follows: 1– Quercetin-3-rutinoside (1.3 ± 0.1), 2 – Quercetin-glucoside (6.3 ± 0.7), 3 – Quercetin-arabinoside (1.08 ± 0.04), 4 – Quercetin-3-rhamnoside (49 ± 2), 5 – Kaempferol-3-glucoside (70 ± 12), 6 – Kaempferol-arabinoside (6.4 ± 0.5), 7 – Kaempferol-rhamnoside (64 ± 8), 8 – Quercetin (160 ± 26), 9 – Kaempferol (146 ± 5).
